# The mitochondrial genome of a giant water bug *Lethocerus deyrollei* (Hemiptera: Belostomatidae) from South Korea

**DOI:** 10.1080/23802359.2021.1893616

**Published:** 2021-03-19

**Authors:** Eun Hwa Choi, Su Youn Baek, Ashraf Akintola, Bia Park, Jihye Hwang, Gyeongmin Kim, Cho Rong Shin, Ui Wook Hwang

**Affiliations:** a Department of Biology Education, Teachers College & Institute for Phylogenomics and Evolution, Kyungpook National University, Daegu, South Korea; b Institute for Korean Herb-Bio Convergence Promotion, Kyungpook National University, Daegu, South Korea; c Biomedical Convergence Science and Technology, Kyungpook National University, Daegu, South Korea; d School of Life Sciences, Graduate School, Kyungpook National University, Daegu, South Korea

**Keywords:** A giant water bug, *Lethocerus deyrolli*, mitochondrial genome, an endangered species, South Korea

## Abstract

A giant water bug *Lethocerus deyrollei* (Hemiptera: Belostomatidae) is a large, predatory, and nocturnal hemipteran insect, which has been considered threatened and thus enrolled as an endangered species in South Korea and Japan. Here, we characterized the complete mitochondrial genome of *L. deyrollei*, which has a circular form with 19,295 bp in length, which is the longest when compared to those of the 111 hemipteran species reported so far. Its longest genome size is due to the extremely extended CR (4686 bp), which is much longer than those of China and Japan. It consisted of a total of 37 genes (13 PCGs, 22 tRNA genes, and two rRNA genes) and one control region (CR). The genome composition and gene order were identical to those previously reported from the same species of China and Japan with over 99.7% sequence similarities except for CR and *trnI*. The nucleotide composition was highly A + T biased, accounting for 71% of the whole mitochondrial genome, as in other species of Nepoidea. Based on the aa sequences of 13 PCGs, we reconstructed a maximum likelihood tree, which indicated that the three mitochondrial genomes of *L. deyrollei* from South Korea, China, and Japan are grouped, and also *Lethocerus*, Belostomatidae, Nepoidea, Nepomorpha, Heteroptra are strong monophyletic groups, respectively.

The giant water bug *Lethocerus deyrolli* (Vuillefroy, 1864) is a large, nocturnal, and aquatic insect belonging to the family Belostomatidae (Order Hemiptera). It is one of the principal predators in a wide range of freshwater habitats, which includes rice fields, ponds, lakes, and slow-flowing rivers (Mukai et al. 2005). It is known that they are distributed in South Korea, Japan, East China, East Indochina, and the Amur region of Russia (Perez-Goodwyn 2006). They were found very common in the distributed area, but have recently declined drastically in South Korea and Japan. In these regions, thereby, they have been considered threatened and thus enrolled as an endangered species (Ohba 2007; National Institute of Biological Resources (NIBR) 2018). In the case of South Korea, it has been denoted as a second-class endangered species by the Ministry of Environment in 2007 due to the drastic reduction in population size and distribution: only a few populations remain in specific natural habitats in the coastal areas of Gyodong Island and majorly in Jeju Island (Jo 2003). For their conservation and management purposes, characterization of mitochondrial genome information of endangered species is of importance because of the usefulness for elucidating their genetic diversity, genetic structure, and molecular evolutionary history (Hwang and Kim 1999; Shin et al. 2015; Jang and Hwang 2016; Jang et al. 2016; Kim et al. 2019).

Here, we attempted to characterize the complete mitochondrial genome of *L. deyrollei* collected from South Korea, with which we examined the phylogenetic position within the superfamily Nepoidea. *Lethocerus deyrollei* specimens were collected from Eoeum-ri, Aewol-eup, Jeju city, Jeju-do (33°22'51.6"N 126°21'09.0"E), South Korea, with a landing net on the 4 August 2015. Specimens were stored in 100% ethanol and deposited under the voucher number GEIBIN0000339415 in the National Institute of Biological Resources (NIBR), Total genomic DNA was isolated from tissues using a DNeasy Blood and Tissue Kit (QIAGEN Co., Germany) following the manufacturer’s protocol, and the mitochondrial genome was amplified by long-range PCR method using Expand^TM^ Long Template PCR System (Roche Co., Germany). In total, one universal and three species-specific primer sets were used in this study (Supplementary table). The PCR products were purified using a QIAquick PCR Purification Kit (QIAGEN Co., Germany) and then sequenced using an ABI Prism 3730 DNA sequencer (PerkinElmer, USA) with a BigDye Termination Sequencing Kit. The obtained sequences were aligned and trimmed using the Clustal X2 program (Larkin et al. 2007) and BioEdit 7.0.9 program (Hall 1999). Protein-coding genes (PCGs), rRNAs, tRNAs, and a control region were characterized using NCBI Basic Local Alignment Search Tool (BLAST) and a program tRNAscan-SE (Chan and Lowe 2019). For phylogenetic analysis, Clustal X2 (Larkin et al. 2007) was used in aligning the PCG amino acid sequences. With separate amino acid sequence alignments of the 13 PCGs across 16 hemipteran species, we constructed a concatenated alignment set (3301 aa). Using the maximum likelihood (ML) method, the phylogenetic tree was built with 1000 bootstrap replicates and substitution model mtART + F + I + G4, through IQ-TREE webserver (Trifinopoulos et al. 2016).

According to the results, the complete mitochondrial genome of *L. deyrollei* is a circular form (19,295 bp in length; GenBank accession no. KU237288), which is revealed to be the longest when compared to those of the 111 hemipteran species reported to date. The mitochondrial genome includes 37 genes (13 PCGs, 22 tRNAs, and 2 rRNAs) and one control region (CR). Out of the 37 genes, 26 are located on the heavy (+) strand and 11 on the light (–) strand. We compared the result with those published from the same species of China (Li et al. 2017) and Japan (Nakasako et al. 2020). The longest hemipteran genome size shown in the mitochondrial genome of *L. deyrollei* is due to the extremely extended CR which is 4686 bp in length. The CRs shown in the Chinese and Japanese *L. deyrollei* genomes are much shorter than that of South Korea: 775 bp for China and 972 bp for Japan. Except for CR and *trnI* (not found in the Chinese one), it showed that these mitochondrial genomes contain high nucleotide sequence similarity: 99.73% between South Korea and China, 99.82% between South Korea and Japan, and 99.74% between China and Japan. The base composition of the entire mitochondrial genome of *L. deyrollei* exhibits a typical A + T biased pattern (71.1%), which is similar to those of the superfamily Nepoidea, including *Lethocerus indicus* (70.5%, NC_017294), *Diplonychus rusticus* (69.8%, Hua et al. 2009), *Nepa hoffmanni* (72.0%, Zhang et al. 2016), and *Laccotrephes robustus* (70.6%, Hua et al. 2009). The ML tree was reconstructed based on the amino acid sequences from 13 PCGs of the *L. deyrollei* mitochondrial genome with those of 16 representative hemipteran species. The result showed that *L. deyrollei* collected from South Korea is grouped with the same species from China (Li et al. 2017), and Japan (Nakasako et al. 2020), having a sister of *Lethocerus indicus* (Figure 1). The family Belostomatidae (including *L. deyrollei*) and Nepidae within the monophyletic superfamily Nepoidea strongly formed monophylies, respectively (BP 100). Besides, the monophylies of Heteroptera (BP 100) and Nepomorpha (BP 88) were robustly supported on the tree, respectively.

**Figure 1.**
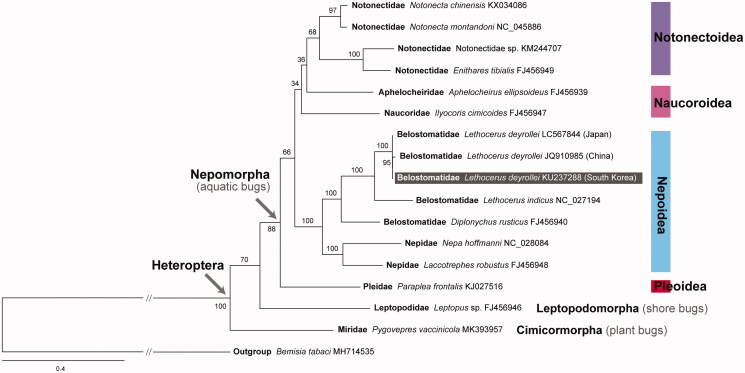
Phylogeny of Heteroptera based on the 13 PCG amino acid sequences of mitochondrial genomes under the mtART + F + I + G4 model inferred from IQTREE analyses. The mitochondrial genome of *L. deyrollei* obtained in this study is highlighted with a black box. Numbers at nodes indicate bootstrap support values (BP). A silver leaf whitefly *Bemisia tabaci* was employed as an outgroup.

## Data Availability

The data that support the findings of this study are openly available in NCBI GenBank database at (https://www.ncbi.nlm.nih.gov/nuccore/KU237288.1/), which permits unrestricted use, distribution, and reproduction in any medium, provided the original work is properly cited. The information of the supplementary table was deposited in Figshae DB (https://doi.org/10.6084/m9.figshare.13515194.v2).
